# Eradication of Human Ovarian Cancer Cells by Transgenic Expression of Recombinant *DNASE1, DNASE1L3, DNASE2,* and *DFFB* Controlled by *EGFR* Promoter: Novel Strategy for Targeted Therapy of Cancer

**DOI:** 10.4172/2157-7412.1000152

**Published:** 2013-07-21

**Authors:** Marek Malecki, Jessica Dahlke, Melissa Haig, Lynn Wohlwend, Raf Malecki

**Affiliations:** 1 PBMEF, San Francisco, CA 94105, USA; 2 NMRFM, NIH, Madison, WI 53706, USA; 3 UW, Madison, WI 53706, USA; 4 SFSU, San Francisco, CA 94132, USA

**Keywords:** Cancer of the ovary, Epidermal growth factor receptor, Nuclear localization signal, DNase, Apoptosis, Suicide gene therapy of cancer, Personalized therapy of cancer

## Abstract

**Introduction:**

Ovarian cancer is the most deadly among all gynecological cancers. Patients undergoing systemic therapies of advanced ovarian cancers suffer from horrendous side effects. Cancer survivors and their offspring suffer from iatrogenic consequences of systemic therapies: genetic mutations. The ultimate goal of our work is development of therapies, which selectively and completely eliminate cancer cells, but do not harm healthy cells. An important consideration for attaining this goal is the fact that ovarian cancer cells over-express EGFR or its mutants, what becomes the factor discriminating them from healthy cells - a potential facilitator of personalized therapy.

**Specific aim:**

The specific aim of this project was threefold: (1) to bioengineer suicide genes’ carrying vectors guided by synthetic antibodies for EGFRvIII and EGFR; (2) to genetically engineer DNA constructs for the human, recombinant *DNASE1, DNASE1L3, DNASE2,* and *DFFB* controlled by the *EGFR* promoter; (3) to selectively eradicate ovarian cancer cells by intranuclear targeting of the transgenically expressed recombinant DNases.

**Methods:**

Synthetic antibodies for EGFR and EGFRvIII were selected from the human library and used to bioengineer biotag-guided transgenes’ vectors. Coding sequences for the human *DNASE1, DNASE1L3, DNASE2, DFFB* controlled by the *EGFR* promoter were amplified from the human cDNA and genetically engineered into the plasmid constructs also coding for the fusions with NLS and GFP. The vectors carrying transgenes for the DNases were delivered *in vitro* into human ovarian cancer cells from ascites and cultures.

**Results:**

Synthetic antibody guided vectors delivered the transgenes for the recombinant DNases efficiently into the ovarian cancer cells. Transgenic expression and nuclear targeting of the DNases in those cells resulted in destruction of their genomes and led to their death, as validated by labeling with the molecular death tags. In healthy cells, which did not over-express *EGFR*, no changes were recorded.

**Conclusion:**

Targeted expression of the recombinant *DNASE1, DNASE1L3, DNASE2, DFFB* in the ovarian cancers *in vitro* resulted in their complete eradication, but had no effects upon the healthy cells. This novel therapeutic strategy has a potential for streamlining it into *in vivo* trials, as personalized, targeted therapy of ovarian and other cancers.

## Introduction

Ovarian cancer is the most deadly among all gynecological neoplasms with 19% five year survival of patients, diagnosed at advanced clinical stages, in the USA in 2012 [1,2]. Most ovarian cancers originate from epithelial tissue, but embryonal carcinomas are particularly malignant [3–9]. Cancer stem cells contribute to malignancy and developing resistance to therapy [10–14]. Ovarian cancers grow in abdominal cavity without giving specific symptoms, therefore 63% of ovarian cancers are diagnosed only after progressing to advanced clinical stages [1,2,15–17]. Metastasizing cancer cells are often detected in peritoneal washings or ascites [18,19]. Distant metastases to spine or brain are particularly difficult to diagnose and cure [20–22].

Over-expression of the *EGFR* gene is frequent in ovarian cancers [23–31]. While in some studies, *EGFR* mutation deletion variant type III was reported in 92% of the ovarian cancers at the FIGO clinical stage III, in other investigations this mutation was not revealed at all. Although, regulation of this gene’s expression is not yet explained, its promoter is sequenced as absent of TATA and CAAT boxes, with determined transcription start site (TSS) and specificity protein 1(SP1) binding sites [32–37].

Advanced stages of ovarian cancers require systemic therapies, which are unfortunately charged with very poor therapeutic record [1,2,38–40]. Moreover, patients undergoing systemic therapies, including radiation, immuno-radiotherapy, and chemotherapy suffer from horrendous side effects, which range from emesis to tissue damage. Additional harms, inflicted upon survivors and their offspring, are iatrogenic consequences of systemic therapies, which extend far beyond their completion: potential mutations in genomes of the ova, which may lead to infertility of women or congenital diseases of their children [41–60].

Many different cancer therapy modalities exert their effects by triggering apoptotic or necrotic cascades. These include triggering of multiple signaling pathways, cytochrome release, initiating oxidative stress, and/or activation or transgenic expression of caspases. As the grand finale, DNases execute destruction of genomic DNA, which leads to cells’ death. However, cancer cells develop mechanisms, which expel therapeutics, counteract activation of caspases, and reverse apoptotic processes, which help them to avoid death [61–76]. Aforementioned phenomena prompted our research on targeted cancer cell suicide inducing therapies [77–81]. Our plan was to bioengineer therapeutics targeted closer to their effectors along signaling pathways. This should reduce options for death cascades’ reversals. The most direct induction of cancer cell suicide, we have attained by genetic engineering and transgenic expression of recombinant, human DNases in cancer cells of ovaries and testes [80].

The ultimate goal of our work was development of therapy, which would selectively eliminate ovarian cancer cells, but would not harm healthy cells. Realistic routes for attaining this goal started to shape up, when we bioengineered synthetic antibody guided vectors carrying multiple transgenes and genetically engineered DNA constructs for human recombinant DNases targeted into cells’ nuclei [8,9,77,80–84].

## Specific Aim

The specific aim of this project was threefold: (1) to bioengineer suicide genes’ carrying vectors guided by synthetic nano-antibodies for EGFR and EGFRvIII; (2) to genetically engineer DNA constructs for the human, recombinant *DNASE1, DNASE1L3, DNASE2,* and *DFFB* controlled by the *EGFR* promoter; (3) to selectively eradicate ovarian cancer cells by intranuclear targeting of the expressed transgenic DNases.

## Methods

### Synthetic antibodies for EGFR and DNA

Synthetic nano-antibodies against EGFRvIII and EGFR were bioengineered as described earlier and the sequences were published [8,80–86]. Briefly, fresh blood was received from the cancer patients with the Institutional Review Board (IRB) approval and with the Informed Consent Forms (ICF) signed. White blood cells (WBC) were isolated using Ficoll-Hypaque technique. The B cells were isolated using genetically engineered antibodies targeting CD19 and CD20. The total mRNA was isolated using Trizol reagent (Molecular Research Center, Inc. Cincinnati, OH). The cDNA was generated using random hexamers (Intergrated DNA Technologies, Coralville, IA) and reverse transcriptase (Promega, Madison, WI) in reactions involving denaturing RNA at 70°C followed by reverse transcription carried at 42°C for 15 min. The cDNA quality was tested by the polymerase chain reaction (PCR) of beta actin and GAPDH as reference genes with the commercially available primers (ABI, Foster City, CA). For amplification of variable fragments, the primers sets were designed using the Kabat’s database. They were synthesized on the 380A DNA Synthesizer (ABI, Foster City, CA). The variable fragments were amplified by polymerase chain reaction using the mix of the generated cDNA, the synthesized primers, dNTPs, and Taq DNA polymerase (Hoffmann–La Roche, Basel, Switzerland) using the Robocycler (Stratagene, San Diego, CA) or Mastercycler (Eppendorf, New York, NY). The blunt ended amplicons were inserted into the pM construct containing the single EGFR transmembrane sequence imported from the GenBank Reference Sequence ID: NM_005228 in Public Domain (NCBI, Bethesda, MD). The DNA plasmid constructs also contained metal binding domains capable of chelating superparamagnetic and fluorescent metals as described [9,77]. The constructs were electroporated and expressed in human myelomas. All the expressed clones were labeled in liquid phase with the free transgenic receptors, which were modified with fluorescent or superparamagnetic reporters. The clones expressing the heavy (HC) and light chains (VL) were selected on the fluorescent activated cell sorter FACS Calibur (Becton-Dickinson, Franklin Lakes, NJ) or magnetic activated cell sorter (MACS) (the sorter built based upon the grants from the NSF for Dr Malecki, Principal Investigator). The new constructs were also expressed in human myelomas. The coding sequences were verified after total RNA extraction, reverse transcription, amplification, and sequencing of amplicons on the ABI 3130XL or Junior DNA Sequencer (ABI, Foster City, CA). The clones of the antibodies used to this study were encoded MR24 for the EGFRvIII and MS23 for the EGFR. For the first round of selections, the free, transgenic, soluble, extracellular domains of the receptors were generated as the baits. They were designed based upon the coding sequence for the human EGFRwt based upon the sequence imported as the NCBI Reference Sequence: AC006977.3 and for the human EGFRvIII carrying mutation deletion of the exons 2–7 as described and their sequences were published [8,85,86]. The primers were designed using the Primer Express Primer Express (ABI, Foster City, CA) and synthesized. After amplification and purification, the cDNA for the EGFR or EGFRvIII domains was transduced in myelomas followed by the gene expression products’ purification on HPLC.

Synthetic nano-antibodies against dsDNA single chain variable fragments were bioengineered as described earlier and the sequences were published [9,80]. Briefly, the B cells were selected from the patients suffering from LE. They were sorted with MACS, after the DNA was modified with superparamagnetic antibodies. Alternatively, they were sorted by FACS, after the DNA was tagged with fluorescent reporters. RT PCR was performed on each cell carrying dsDNA targeting variable fragments. Coding sequences for the variable fragments were amplified and cloned within the plasmid vectors and expressed in human myelomas and B cells, as described and with all the sequences published [8,84].

### Cultures of ovarian cancer, epithelial, and bone marrow cells

After performing the surgical biopsy and/or paracentesis, followed by an evaluation by surgical pathologist on site, the cells were collected into the Dulbecco Modified Essential Medium within cell culture flasks. The growing ovarian cancer cultured cells (OCC) were maintained within the cell culture incubators at 37°C, saturated humidity, and mixtures of CO_2_/O_2_/N_2_ gases. The cells expressed 0.03–3 million EGFRwt or EGFRvIII per cell as determined by NMRS after labeling with superparamagnetic antibodies or EDXS after labeling with elemental tagged antibodies. The viability of the cells was determined by labeling with bisbenzimide and propidium iodide cocktail versus fluorescent or superparamagnetic molecular death biotags (Invitrogen, Carlsbad, CA, USA). After labeling with synthetic antibodies against dsDNA and PS, sorting out apoptotic and dead cells was performed on FACS Calibur or FACS Vantage SE (Becton-Dickinson, San Jose, USA) or our own magnetic sorter as described [9,84].

Moreover, the human ovarian epithelial carcinoma line OVCAR3 cells (ATCC, Manassas, VA, USA) and human bone marrow were transfected with the DNA plasmids, which were carrying coding sequences for the truncated version of the *EGFRvIII* controlled by *EGFR* promoter [8,9,84]. For evaluating gene expression through qPCR, the primers and protocols were designed using Primer Express (ABI, Foster City, CA). The qPCR reactions were run on HT7900 (ABI, Foster City, CA). The EGFR strongly expressing cells were used for validating the generated EGFRwt and EGFRvIII antibodies. Control apoptosis was induced with 30 μM C2-ceramide and 0.3 μg/ml actinomycin D or 2 μM staurosporine for 6 h. Control patterns of digestion were prepared by permeabilization of the cells with 0.1% NP40 and digestion with the four hrDNases.

### Bioengineering vectors for human *DNASE1, DNASE1L3, DNASE2, DFFB* controlled by *EGFR* promoter. Studying effects of transgenic expression of DNases

Tissue was obtained from cancer free margins during surgery of patients suffering from cancers of pancreas, liver, and ovary. Genomic DNA was isolated using Nucleic Acid Extractor Model 340A (ABI, Foster City, CA). Total mRNA was isolated using Trizol reagent (Molecular Research Center, Inc. Cincinnati, OH). The cDNA was generated using random hexamers (Intergrated DNA Technologies, Coralville, IA) and reverse transcriptase (Promega, Madison, WI). The following coding sequences were imported from the NCBI and used to design the primers using PrimerBlast: homo sapiens deoxyribonuclease I (*DNASE1*): NCBI Reference Sequence: NC_000016.9; homo sapiens deoxyribonuclease 1L3 (*DNASE1L3*): NCBI Reference Sequence: NC_000003.11; homo sapiens deoxyribonuclease II (*DNASE2*): NCBI Reference Sequence: NC_000019.9; homo sapiens DNA fragmentation factor B (*DFFB*): NCBI Reference Sequence: NC_000001.10. The primers were synthesized on the 380A DNA Synthesizer (ABI, Foster City, CA) and the sequences amplified on the Robocycler (Stratagene, San Diego, CA), Mastercycler (Eppendorf, Hamburg, Germany), or 7500, 7900 HT qPCR systems (ABI, Foster City, CA). The following coding sequences were imported from the NCBI and synthesized on the DNA synthesizer: homo sapiens promoter for EGFR (*EGFR*): NCBI Reference Sequence:

NC_000007.13 (TCCTCTCTCGCTGCTCGCGCCTCGGCCCGCGCGAGCTAGACGTCCGGG (Prom_EGFR), homo sapiens nuclear localization signal for nucleoplasmin (*NPM1*) (NLS_NPM1), and short unique tagging sequence [82,83]. The coding sequences for each of the DNases were joined by overlap extension with those for the Prom-EGFR and NLS-NPM. As fluorescent reporters, the following coding sequences for fluorescent proteins within the plasmids were according to: GFP as in GenBank Accession M62653.1 (the gift from Dr D. Prasher) [87,88] and its BFP, CFP, and YFP mutations (the gift from Dr R. Tsien) [89,90]. Alternatively, as superparamagnetic reporters, the sequences harboring for Gd chelators were synthesized [77]. Those sequences were inserted to code carboxyl termini of the expressed fusion proteins. The resulting DNA constructs were amplified and cloned into pUC vector, which contained the Ampicillin resistance and Ori signal for propagation and selection in *E. coli.*

These four DNases were assembled into the transfection vectors, which were bioengineered as described [8]. Briefly, the synthetic antibodies against DNA were carrying biotin tags at the carboxyl termini. After binding the DNA constructs for the DNases, they were docked into the biotin binding site of the recombinant avidin one at a time to create the DNA non-viral vectors. These vectors were guided by synthetic biotags into the cells as described [82–84]. These vectors carrying plasmids of the same sizes but with reversed direction coding sequence for DNases or without NLS were delivered as the controls. Since, the biotags carried permanent fluorescent, radionuclide, or superparamagnetic reporters, efficacy of targeted delivery was easy to quantify with MPFS, EDXS, GRS, or NMRS.

Effects of transgenes’ expression were determined by MPFS of living cells’ chromatin and electrophoresis of nuclei’s lysates. Surfaces of cryo-immobilized cells were studied by FESEM. Architecture of nuclear chromatin was revealed by EFTEM. Apoptotic and necrotic cells were quantified after labeling with synthetic antibody based biotags against dsDNA and against PS. These biotags were rendered fluorescent or superparamagnetic, so that quantification of dead or apoptotic cells was pursued with FCM, XRFS, or NMRS.

### Flow cytometry (FCM), Fluorescently activated cell sorting (FACS), Ploem’s and Multiphoton Fluorescence Spectroscopy (MPFS)

The cell clusters were thoroughly disintegrated into single cell suspension by short treatment with the PIPES buffered DNase, RNase, trypsin, and collagenase as described [8,9]. The negative selection involved depletion of apoptotic cells with the fluorescent or magnetic antibodies anti-PS and the dead cells with the antibodies anti-DNA to reach above 99.5% of purity. The enriched populations were measured with the Calibur, *Vantage SE, or Aria* (Becton-Dickinson, Franklin Lakes, NJ, USA) or the FC500 (Beckman-Coulter, Brea, CA, USA). The fluorescently labeled cells were imaged with the Axiovert (Zeiss, Oberkochen, D, EU) equipped with the lasers generating 364 nm, 457 nm, 488 nm, 529 nm lines; Odyssey XL digital video-rate confocal laser scanning imaging system operated up to 240 frames/s under control of Intervision software (Noran, Madison, WI, USA), and the Diaphot (Nikon, Tokyo, Japan) equipped with the Microlase diode-pumped Nd:YLF solid state laser (1048 nm line), the pulse compressor with the pulses’ rate 300 fs at 120 MHz and the MRC600 scanning system under control of Comos software (the multi-photon fluorescence station built with the grant from the NIH for Dr J. White, Principal Investigator). Deconvolution of images was done on the Indy workstation (Silicon Graphics, Fremont, CA, USA).

### Nuclear Magnetic Resonance Spectroscopy (NMRS); Magnetically Activated Cell Sorting (MACS)

The cells were labeled for positive selection with the superparamagnetic antibodies against EGFRvIII or EGFR and for the negative selection with the superparamagnetic antibodies against dsDNA and PS. The small aliquots were dispensed into the magnetism-free NMR tubes (Shigemi, Tokyo, Japan). The relaxation times T1 were measured in resonance to the applied pulse sequences on the NMR spectrometers: DMX 400 WB or AVANCE II NMR (Bruker, Billerica, MA) or the Signa clinical scanners (GE, Milwaukee, WI, USA). The superparamagnetic Fvs were also used to isolate the labeled cells from the solution using the magnetic sorter to reach above 99.5% of purity (the sorter designed and built based upon the NSF funds – PI: Dr M. Malecki).

### Electron Energy Loss Spectroscopy (EELS); Energy Dispersive X-Ray Spectroscopy (EDXS); X-ray Reflection Fluorescence Spectroscopy (XRFS)

The samples, which were cryo-immobilized, presented the life-like supramolecular organization. Molecular imaging was pursued as described [77]. The field emission, scanning transmission, electron microscope FESTEM HB501 (Vacuum Generators, Kirkland, WA, USA) was equipped with the energy dispersive x-ray spectrometer (EDXS) (Noran, Middleton, WI, USA) and post-column electron energy loss spectrometer (EELS) (Gatan, Pleasanton, CA). The cryo-energy filtering transmission electron microscope 912 Omega was equipped with the in-column, electron energy loss spectrometer (EELS) (Zeiss, Oberkochen, D, EU). The cryo-energy filtering transmission electron microscopes 410 and 430 Phillips were equipped with the post-column, electron energy loss spectrometers (EELS) (Noran, Middleton, WI, USA). The field emission, scanning electron microscope SEM1530 (Zeiss, Oberkochen, D, EU) was equipped with the energy dispersive x-ray spectrometer (EDXS) (Noran, Middleton, WI, USA). The field emission, scanning electron microscope 3400 was equipped with the energy dispersive x-ray spectrometer (EDXS) (Hitachi, Tokyo, Japan). The images and spectra were acquired using the ccd camera operating under the image acquisition and processing software (SIS, Herzogenrath, D, EU or Emispec Systems, Tempe, AZ, USA). In this study, the ICP standard of 1000 mg/l of mono-element Gallium (CPI International, Denver, CO, USA) was added to 500 microL of each sample to the final concentration of 10 mg/l. The data were generated from the S2 Picofox TXRF spectrometer equipped with a molybdenum (Mo) X-ray target and the Peltier cooled Xflash Silicon Drift Detector (Bruker AXS, Fitchburg, WI, USA). Scan times ranged up to 1000 seconds. The automatic sample changer, which can hold up to 25 samples, was also used along with the SPECTRA 7 software for the instrument control, data collection, and analysis (Bruker AXS, Fitchburg, WI, USA). For elemental analysis TRXF was applied.

### Immunoblotting (IB)

The cells and tissues were either frozen in liquid nitrogen, crushed, and thawed or disintegrated with ultrasonicator (Branson Ultrasonic, Danbury, CT, USA) within the sample buffers for native protein analysis. They were stored in liquid nitrogen or electrophoresed in the native buffer (Invitrogen, Carlsbad, CA, USA). They were vacuum or electro-transferred onto the PVDF membranes (Amersham, Buckinghamshire, UK, EU). The membranes carrying the transferred proteins were soaked within human serum and labeled with the antibodies. Purified receptors and DNases were the controls. The images of the blots were acquired and quantified with Fluoroimager (Molecular Dynamics, Sunnyvale, CA, USA), Storm 840 (Amersham, Buckinghamshire, UK, EU), and Odyssey (Li-Cor, Lincoln, NE, USA).

### Quantitative Reverse Transcription and Polymerase Chain Reaction (qRTPCR)

To determine concentrations of transcripts of EGFR and EGFRvIII, total isolated mRNA served as the template to generate cDNA through reverse transcription using random hexamers and reverse transcriptase (ABI, Foster City, CA, USA) as described [8,9]. The cDNA sequence was imported from the NCBI under the Reference Sequence: NM_005228.3, the primers designed using Primer Express (ABI, Foster City, CA, USA) or as those described earlier [8,9,84]. The primers for beta actin and GAPDH served as the reference genes (ABI, Foster City, CA, USA). They were all synthesized on the 380A DNA Synthesizer (ABI, Foster City, CA). Amplifications were carried using the mix of the cDNA, the synthesized primers, dNTPs, and Taq DNA polymerase (Hoffmann–La Roche, Basel, H) on the Robocycler (Stratagene, San Diego, CA, USA), Mastercycler (Eppendorf, Hamburg, D, EU), or 7500, 7900 systems (ABI, Foster City, CA, USA) as described [8,9]. The images of the gels were acquired and quantified with Fluoroimager (Molecular Dynamics, Sunnyvale, CA, USA) or Storm 840 (Amersham, Buckinghamshire, UK, EU). The levels of the transcripts were all normalized against GAPDH or actin, and thereafter calculated as the ratios between the transcript concentration in the examined patient’s cells versus the cells from the healthy control tissues and cultures.

To distinguish splicing variants and mutants, the genomic DNA was isolated as described [8,9,84]. The sequencing was performed on the DNA Sequencer ABI 3130XL (ABI, Foster City, CA) and Junior (Roche, San Diego, CA).

## Statistical Analysis

The statistical analysis and presentations were performed using GraphPad Prism software (GraphPad Software, Inc, San Diego, CA, USA). All the data were acquired from at least three independent runs of each patient’s or cell culture samples. For comparisons between two groups of data, the unpaired t-test was applied and *P* calculated. The statistics were calculated and presented as mean ± standard deviation of the mean. The results were considered as statistically significant for *P* < .05.

## Results

**Figure 1:**
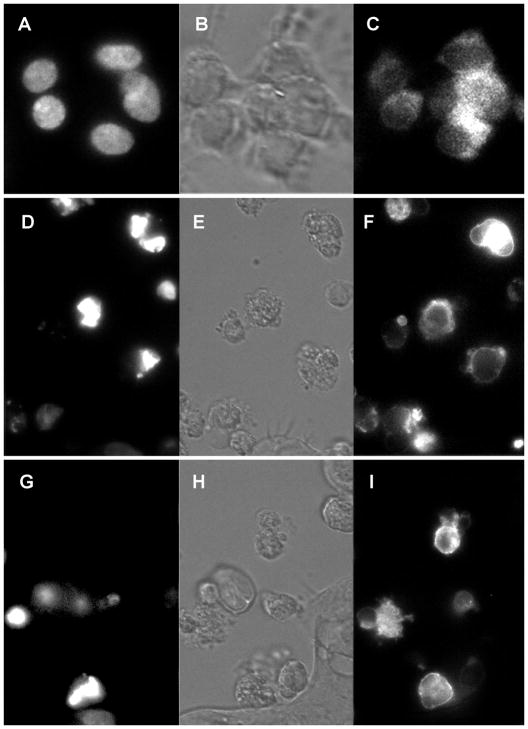
The DNA constructs with the coding sequences for human recombinant DNase1, DNase1L3, DNase2, DFFB (hrDNases) were delivered by anti-EGFRvIII (DEF) and anti-EGFR (GHI) antibody guided vectors into the nuclei of EGFRvIII + or EGFR+ over-expressing ovarian cancer cells from the patients’ ascites. They were labeled with the anti-dsDNA (D,G) or anti-phosphatidylserine (F,I) and imaged with phase contrast (E,H) or Ploem’s epifluorescence (D,F,G,I). Non-transduced EGFRvIII+ ovarian cancer cells from ascites stained with bisbenzimide (A) and labeled with anti-EGFRvIII antibody (C), were imaged with phase contrast (B) or Ploem’s fluorescence (A,C) as the controls. Transgenic expression and intranuclear targeting of the hrDNases in ovarian cancer cells resulted in collapse of the chromatin architecture, as highlighted after labeling with anti-dsDNA fluorescent antibodies (E,G), as well as externalization of phospatidylserine as highlighted after labeling with anti-PS fluorescent antibodies (F,I).

**Figure 2:**
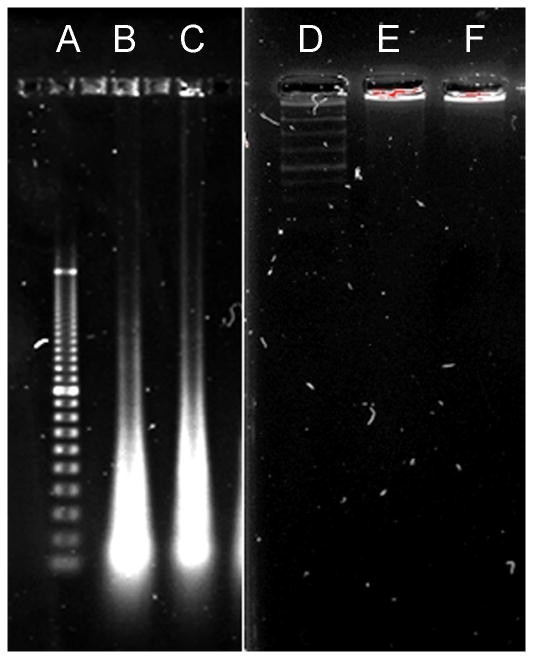
The DNA constructs with the coding sequences for human recombinant DNase1, DNase1L3, DNase2, DFFB (hrDNases) were delivered by anti-EGFRvIII (B,E) and anti-EGFR (C,F) antibody guided vectors into the nuclei of EGFRvIII + or EGFR+ over-expressing ovarian cancer cells from the patients’ ascites. Genomic DNA from these cells was isolated, electrophoresed, and stained. The reference ladders were 100bp (A) and 200bp (D). In the targeted and transduced cells, genomic DNA was completely degraded (B,C). In the cells, which were transduced with the reversed orientation vectors (E) or non-transfected (F), genomic DNA was retained in the loading wells.

**Figure 3:**
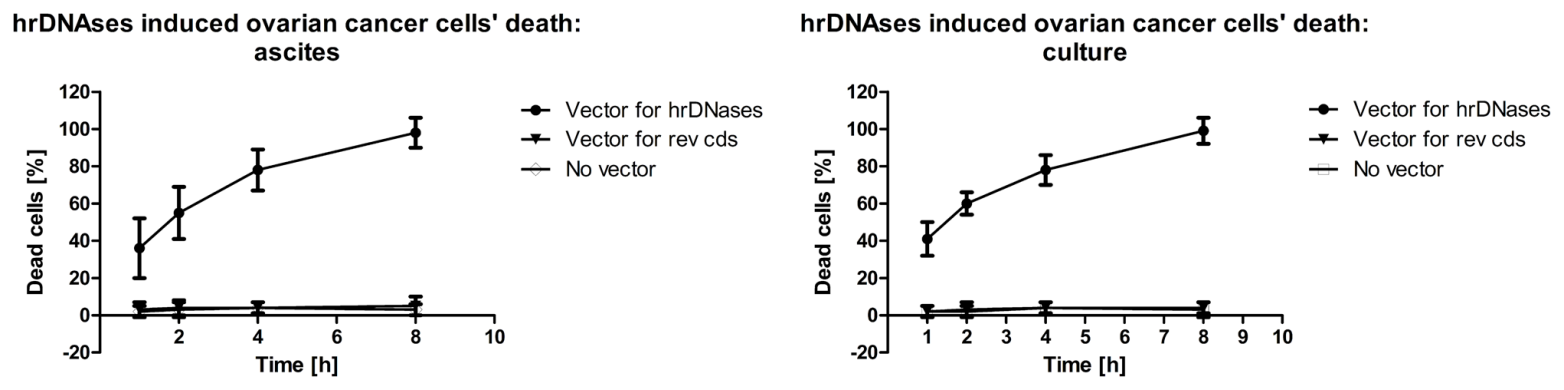
Deadly effects of transgenic expression of DNases in the ovarian cancer cells over-expressing EGFRvIII from ascites and culture were quantified by labeling of the cells with superparamagnetic antibodies against dsDNA followed by measuring relaxivities with NMRS. That followed by separation of the labeled cells with MACS. Lethal effects of transgenic DNases onto the ovarian cancer cells were quantified by labeling with the elemental tagged antibodies for EDXS to yield identical results. Viability of the cells remained unaffected, when the cells were transfected with the vectors coding reversed orientation sequences as compared to the cells not exposed to any vectors at all.

**Figure 4:**
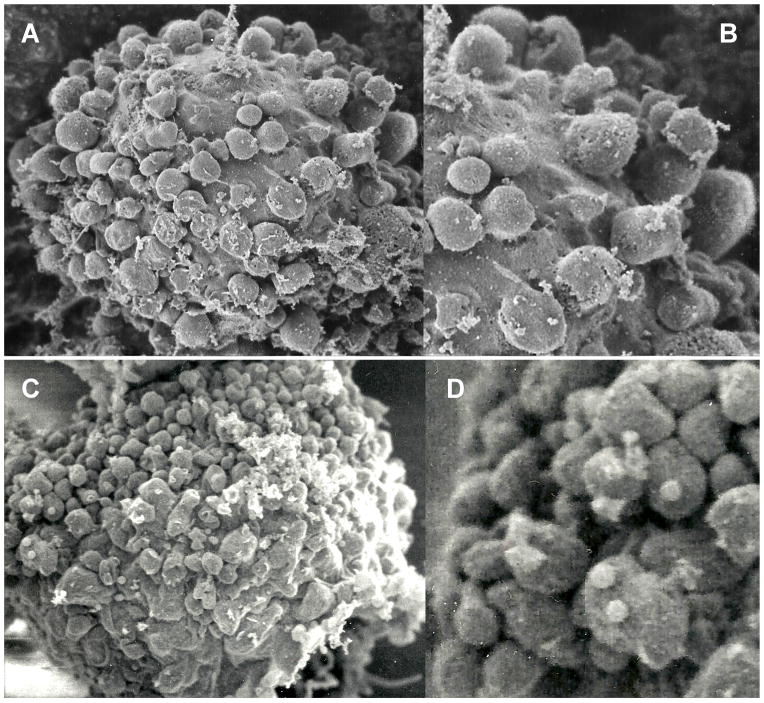
Transduction of the EGFRvIII+ over-expressing ovarian cancer cells with the DNA constructs for the human recombinant DNase1, DNase1L3, DNase2, DFFB (hrDNases) resulted in their surfaces’ topographies disfigured by multiple blebs (C,D). These could be compared for the surface blebs, which occurred as the results of ROS-induced apoptosis in the cultured OVCAR cells (A,B). Pores in the membranes’ blebs, which are only seen at seen at high magnifications, are the routes of entry for labels targeting their content. Presence of these pores in the blebs’ membranes explains observations that the blebs’ contents are labeled long before the cells’ interiors. HFW A,C: 20 μm; B,D: 7.2 μm.

**Figure 5:**
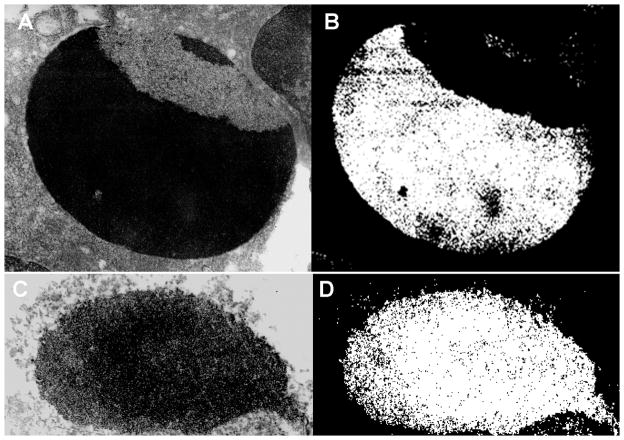
Transduction of the EGFRvIII+ over-expressing ovarian cancer cells with the DNA constructs for the human recombinant DNase1, DNase1L3, DNase2, DFFB (hrDNases) resulted in complete destruction of their chromatin architecture (C,D). This could be compared with the state of collapse of chromatin architecture, which occurred as the results of ROS-induced apoptosis (A,B). In the rapidly cryo-immobilized EGFRvIII+ overexpressing cultured ovarian cancer cells, which were labeled with the anti-dsDNA superparamagnetic antibodies, chromatin architecture was revealed by EFTEM with the filter at the zero loss energy and contrast tuning (A) and distribution of the genomic DNA by ESI with the filter set at the Gd edge (B) [Malecki et al. 2013. WO2012048161 http://patentscope.wipo.int/search/en/WO2012048161]. HFW: 11.25 μm.

We have determined the effects of transgenic expression of the genes for the human, recombinant DNases (hrDNases) on human ovarian cancer cells from ascites of patients with ovarian cancers and from cultures by six different strategies: imaging of genomic DNA (gDNA) architecture and phosphatidylserine (PS) externalization in living cells; evaluating integrity of genomic DNA (gDNA) on gels; nuclear magnetic resonance spectroscopy (NMRS) of the cells labeled with superparamagnetic antibodies against dsDNA and PS; energy dispersive x-ray spectroscopy (EDXS) of the cells labeled with elemental tags; field emission scanning electron microscopy (FESEM) of surface topography on the rapidly cryoimmobilized cells; and field emission energy filtering transmission electron microscopy (FEEFTEM) of the chromatin architecture in the cells’ nuclei (Figures 1–5). We have compared these effects with those observed, while exerted upon cells in control groups: bone marrow, ovarian tissue, and glioblastomas. We have validated the effects of the expression of the transgenic DNA by transfecting all those cells with the vectors carrying the coding sequences for the hrDNases in reversed orientation.

Having applied the first strategy, we have determined the effects of the hrDNases upon overall architecture of the genomic DNA by bis-benzimide (Hoechst) and viability by propidium iodide (PI) staining for fluorescence microscopy and flow cytometry (Figure 1). The EGFvIII and EGFR over-expressers were selected by fluorescent (FACS) or magnetic activated sorting (MACS). Three rounds of assays were performed. The images are representative to all acquired. Synthetic nano-antibodies, which guided the vectors, demonstrated high specificity and exquisite sensitivity [8]. The anti-EGFRvIII and anti-EGFR antibody guided vectors delivered the DNA constructs for DNase1, DNase1L3, DNase2, DFFB into the nuclei of the human EGFRvIII and EGFR over-expressing ovarian cancer cells from ascites and cultures. Cultured healthy bone marrow cells served as the negative controls. Cultured EGFRvIII+ glioblastoma cells and transduced EGFRvIII+ OVCAR cells served as the positive controls. The DNA constructs with the reversed coding sequences served as the DNA constructs’ efficacy controls. The cells exposed to apoptosis inducing agents were used as the references of apoptotic patterns of chromatin. The human recombinant DNases were used to determine the DNA degradation patterns. Transgenic expression and intranuclear targeting of DNases in ovarian cancer cells resulted in collapse of the chromatin architecture. It resulted from apparent degradation of the genomic DNA. These degradation patterns were identical as those in the cells undergoing apoptosis and secondary necrosis. Supply of the vectors into incubation media hosting the bone marrow cells did not have any effects on their chromatin architecture. The vectors carrying the DNA plasmids with the reversed coding sequences did not have effects on architecture of the genomic DNA in any of the cells tested.

Second, we have also studied the effects of the hrDNases upon the genomic DNA from the same cells, which we have previously examined by imaging, but by electrophoresis after isolating the genomic DNA from those cells (Figure 2). The genomic DNA was isolated from the ovarian cancer cells from ascites and cultures, as well as from the glioblastomas and bone marrow as the controls. The cells with ongoing induced apoptosis or necrosis served as the positive control. Also the permeabilized cells exposed to the DNases functioned as the controls. The results from three assays were included. The ones presented here reflect all studied. In the cells, in which these transgenic hrDNases were expressed, the genomic DNA was completely degraded. In the cells, which did not accept the EGFR or EGFRvIII antibody guided vectors, fragmentation did not occur. In the cells, which absorbed the vectors carrying the reversed coding sequences, there were no changes in integrity of the genomic DNA, but it remained in the same state of organization as that in living non-transfected cells.

Third, accuracy of the vectors’ delivery and efficacy in inducing cells’ death were quantified with the nuclear magnetic resonance spectroscopy (NMRS) and magnetic activated cell sorting (MACS), fluorescence activated sorting (FACS), and energy dispersive x-ray spectroscopy with spectral scintillation counting (Figure 3). These were all, in particular NMRS and MACS, non-destructive analytical approaches, which ensured retention of the cells’ with high viability. The key factor for our ability to determine efficacy of the vectors’ delivery were their modifications, which involved incorporation of superparamagnetic nanoparticles, fluorochromes, or radionuclides, into non-functional domains of the vectors [84]. That was followed by sorting of the transfected cells; which thus were permanently tagged with the reporting molecules. Each assay was repeated three times and the results were averaged. The onset of apoptosis and progression into the secondary necrosis due to transgenic expression of the hrDNases were quantified by labeling of the cells with superparamagnetic antibodies against phosphatidylserine and dsDNA and measuring the labeled cells’ effects upon changes in the samples’ relaxivities with NMRS. That followed by separation of the labeled cells with MACS. These procedure facilitated purity > 99.5% in the batches of the transfected cells to be used for studying the effects of transgenic expression of the hrDNases.

Fourth, ultrafine and early signs of initialized apoptosis manifested on cell surfaces. Therefore, we resorted to ultrastructural imaging by field emission scanning electron microscopy (FESEM) and energy dispersive x-ray spectroscopy (EDXS) of surface topographies on cryo-immobilized and freeze-dried cells (Figure 4). The study focused on the ovarian cancer cells from ascites and cultures, while it also included the cultured ovarian and bone marrow cells serving as the controls. At least twenty cells from each of the three samples were considered. The ones illustrated herein are representative for all studied. The cells undergoing apoptosis were referenced as the positive controls. The early onset of apoptosis in the ovarian cancer cells expressing the transgenic hrDNases was revealed by rapid disfiguration of cell surfaces’ topography in the form of the cell membranes’ blebs. They were identical as those on surfaces of the cells undergoing induced apoptosis. On the cancer cells expressing EGFR or EGFRvIII, which absorbed the vectors carrying the reversed coding sequences for the hrDNases, there were no changes of the membranes’ topography. Also, on the EGFR-healthy cells, which were immersed in the media containing the vectors for the DNases, there were no changes in the membranes’ topographies.

Fifth and final strategy, the ultimate hallmark of apoptotic death was the collapse of the chromatin architecture. To study this phenomenon, we resorted to ultrastructural imaging of chromatin architecture *in situ* by field emission energy filtering transmission electron microscopy (FEEFTEM) and electron spectroscopic imaging (ESI) on 50 nm thin sections of the cryo-immobilized and freeze-substituted cells (Figure 5). At least twenty cells were imaged from each of the samples. The images illustrate features unique to all studied. The EGFRvIII and EGFR over-expressing ovarian cancer cells, which were transduced with the transgenic hrDNases contained the chromatin architecture in the state of complete collapse and degradation. This appearance was identical to that imaged in the cells undergoing final accords of apoptotic death and secondary necrosis, which served as the positive control. The chromatin architecture was not affected by delivery of the vectors carrying the hrDNases’ with the reversed coding sequences. The well sustained architecture of the ovarian and bone marrow cells served as the negative controls. These appearances were *de facto* ultrastructural refinements of the images of chromatin degradation highlighted with fluorescence imaging recorded in the living ovarian cancer cells treated with the anti-EGFR or anti-EGFRvIII guided vectors for the hrDNases.

## Discussion

Herein, we have described the proof-of-concept for targeted eradication of the ovarian cancer cells in five stages: (I) anti-EGFRvIII or anti-EGFR antibody guided delivery of the vectors carrying the transgenes for the human recombinant *DNASE1, DNASE1L3, DNASE2, DFFB*; (II) expressing these transgenes under the control of *EGFR* promoter; (III) guiding the transgenes’ expression products into the nuclei of the ovarian cancer cells; (IV) complete degradation of the ovarian cancer cells’ genomic DNA; (V) death of the transduced ovarian cancer cells.

The main advantages of this novel therapeutic strategy in selective cancer eradication are: (1) precision, (2) speed, (3) irreversibility, and (4) completeness.

(1) The vectors carrying the transgenes for the hrDNases are guided by the synthetic nano-antibodies. As described earlier, they are uniquely specific in targeting the receptors’ domains [8,9,84]. They are permanently tagged and traceable. Absence of Fc, HC, and LC domains reduces the risks of non-specific binding. Moreover, these synthetic nano-antibodies target mutated receptors, which are uniquely present on cancer cells only. There is no cross-reactivity between these antibodies towards the domains of truncated and wild type receptors. All these unique features translate into minimized risks of delivering the transgenes into bystanders. Targeting specificity of our vectors is much higher than that of some viral vectors, which we tested, e.g., VSV (Malecki et al. unpublished). This is critical for efficient therapy as every vector delivered into a cell is equivalent to an increased copy number of the transgenes; thus increase in gene expression and efficacy of the strategy. (2) Already within hours from transfection, the cancer cells manifest early signs of death. This is very different from small molecules used in the current, conventional pharmaco-therapies of cancer, which indiscriminately enter into all cells. Therefore, in conventional pharmaco-therapy, the main challenge for practicing clinicians is to find the very delicate balance in the dose, which will be high enough to kill more sensitive cancer cells and spare the presumably less sensitive healthy cells [31–60,91–98]. Too low doses will not be effective therapeutically and will have to be applied for long periods of time. High doses will amplify horrendous side effects. Targeted therapy described herein practically eliminates this challenge, as the therapeutics are delivered into the cancer cells only. Therefore, they can be delivered in the doses, which will be effective immediately within hours. (3) Systemic therapies often initiate either resistance to the applied therapeutics or survival of resistant clones. Both phenomena are responsible for remission, i.e., rapid progression of cancerous tumors resistant to therapies. Cancer stem cells are suggested to be responsible for these phenomena [10–14]. Chemotherapeutics often work through triggering apoptosis or necrosis. However, cancer cells often develop mechanisms, which evade those therapies, as long as their genomes are intact, so that they use them for expressing genes needed for resistance to therapeutics. This results in production of enzymes capable of either blocking, or reversal of apoptotic processes, or outright expulsion of therapeutics, e.g., ABC transporters [71–76]. The strategy described herein eliminates this problem. Transgenic expression and targeted delivery of the hrDNases leads to the irreversible degradation of the cancer cells DNA and these cells’ death. (4) Surgeries often leave portions of cancers intact [99,100]. Systemic therapies often lead to cancer remission through clonogenic survival and selection. While the sensitive cells are killed by chemotherapeutics, the others have or/and develop resistance. These phenomena lead to rapidly growing clones of cells, which are resistant to applied therapeutic, which quickly propel cancerous tumors’ progression. Incomplete eradications of the cancer cells result in remissions, which are far more difficult to treat. The targeted therapy described herein resolves this challenge. The transgene vectors guided by the synthetic nano-antibodies reach all the cells expressing the targeted receptors. In this project it was a mutant of EGFR. This mutant is also present in other cancers [23–31]. In this realm, we have efficiently targeted the therapeutic vectors to brain cancer cells (Malecki et al. in prep). Considering cancer cells’ heterogeneity the vectors carrying transgenes for the hrDNases may be bioengineered to be guided by synthetic nano-antibodies targeting other receptors. This strategy ensures the complete eradication of all targeted cancer cells.

The main problems of this strategy are: (1) rarity of receptors unique for cancer cells; (2) interception of vectors by reticulo-endothelial system (RES); (3) immunogenicity.

(1) Mutation deletion variant III in the *EGFR* gene is uniquely specific to the cancers cells only, but absent on the healthy cells [23–31]. It results in the truncated receptor on surfaces of its expressers. This makes the mutated receptor the ultimate target for targeted cancer therapeutics. Unfortunately, there are not many targets, which are similarly unique qualitatively. Although far more difficult in finding the correct doses, quantitative differences in gene expression offer therapeutic possibilities for applying this strategy also. (2) If applied *in vivo*, the vector of this size may become intercepted by the patients’ reticulo-endothelial system. This may reduce its efficacy. If it happens during the *in vivo* trials, the bio-stealth molecules including polyethylene-glycol may have to be applied. (3) The components of the vectors may become immunogenic after multiple applications. Therefore, reaching high therapeutic efficacy already during first applications may be beneficial for avoiding this problem. As with immuno-therapies immuno-suppression may also be applied in clinical trials. Moreover, using neutralizing antibodies, which we described earlier, may help to resolve this problem in clinical trials.

Mechanisms of the cells’ death, which is induced by transgenic expression of the hrDNases, are complex due to the composite nature of the therapeutic cocktail. The DFFB and DNase1L3 most definitely promote the mechanism of the genomic DNA degradation identical to that triggered by apoptotic signaling pathways. That is reflected by the featured hallmarks of apoptosis: collapse of chromatin architecture, internucleosomal cuts, and membrane blebs. The DNase1 promotes the mechanism of degradation that is specific for pancreatic digestion. Finally, the DNase2 promotes the mechanism of degradation, which is specific of that occurring in lysosomes. Each of these DNases has slightly different optimum of efficacy. The idea behind using them all was to ensure that in the changing environment of progressing cancers with areas of necrosis and apoptosis spectrum of DNases may effectively meet these challenges. Altogether, they all contributed to the complete degradation of genomic DNA. The latter mentioned mechanisms may mask early apoptotic patterns caused initially by the former. Moreover, the necrosis secondary to apoptosis also leads to complete degradation of genomic DNA. In natural order of events, the initial stages of death, which is usually induced by external factors and is triggering deadly signaling cascades, by apoptosis and necrosis, can be distinguished. Early stages of necrosis include swollen cell volume, dilation of organelles, ruptured plasma membrane, and spill of intracellular contents. Early stages of apoptosis are characterized by cell externalization of phosphatidylserine, membrane blebs, which are first parts of cell membranes loosing integrity and becoming permeable, collapse of the chromatin architecture. The mechanism of “death from inside”, which is induced by complete degradation of the genomic DNA in the ovarian cancer cells by transgenic expression of the four hrDNases, is integration of all those processes.

## Conclusion

Transgenic expression of the human recombinant genes for the DNAses and intranuclear delivery of the transgenes’ expression products in the human ovarian cancer cells resulted in effective degradation of their genomic DNA, which led to complete eradication of cancer cells with no systemic effects upon healthy cells. This novel therapeutic strategy has a potential for streamlining into clinics, as personalized, targeted therapy of ovarian and other cancers.
